# Supplementary material for: Influence of Soil and Water Conservation Measures on Soil Microbial Communities in a Citrus Orchard of Southeast China
Source: Microorganisms. 2021 Feb 4;9(2):319. doi: 10.3390/microorganisms9020319 (PMC7913868; doi:10.3390/microorganisms9020319)
Supplement: Supplementary file 1 [file microorganisms-09-00319-s001.pdf]

Supplementary information

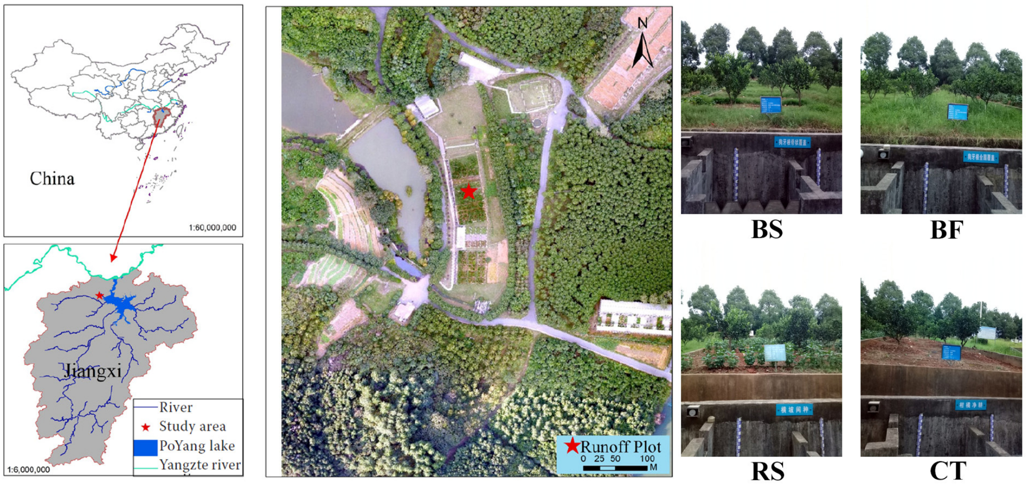

Figure S1. Study area and experimental runoff plots.

BS-Bermuda grass strip intercropping, BF-Bermuda grass full coverage, RS-radish-soybean crop rotation strip intercropping, CT- clear tillage orchards.

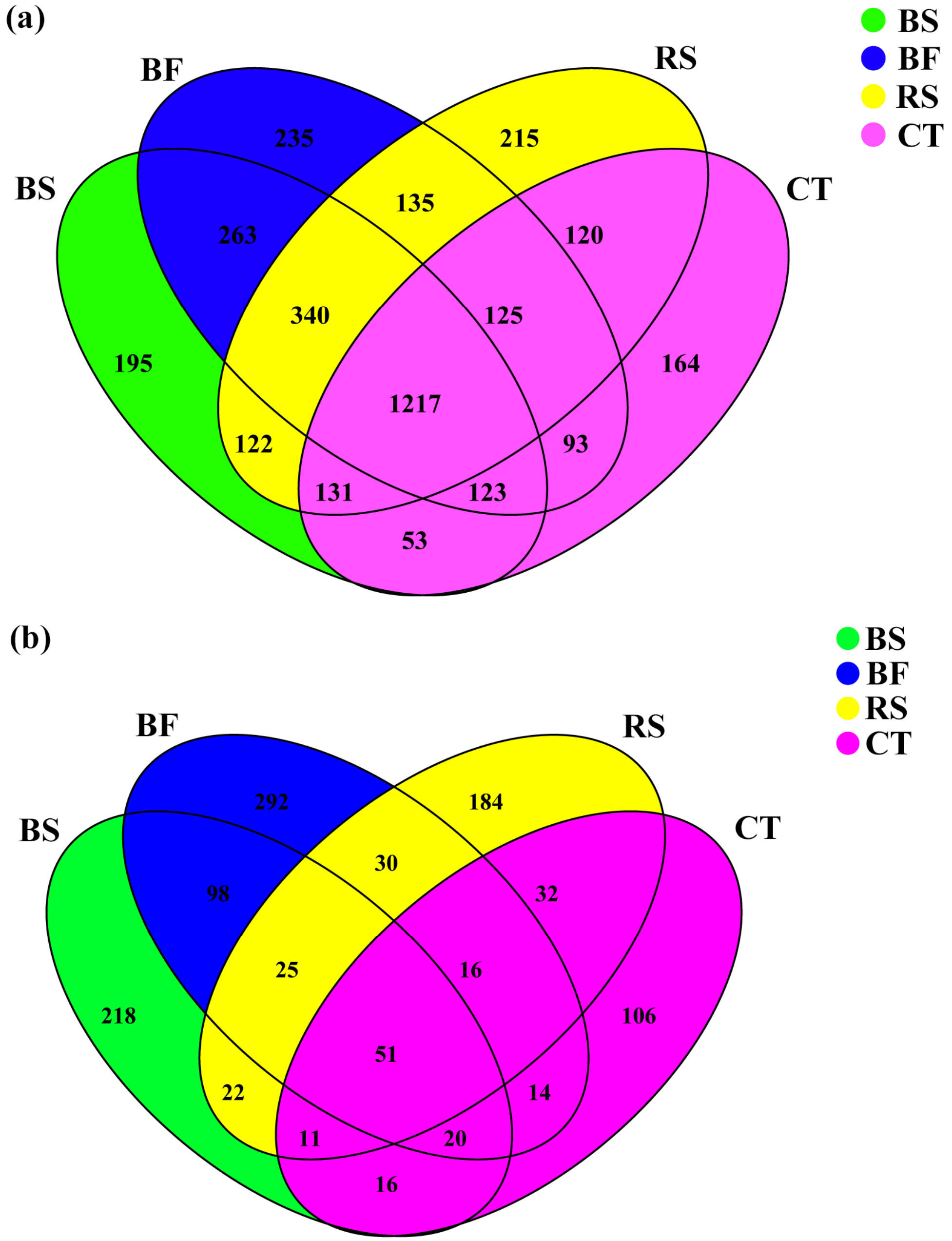

Figure S2. The unique and overlapped OTUs of bacteria (a) and fungi (b) detected in different soil and water conservation measure samples.

(a)

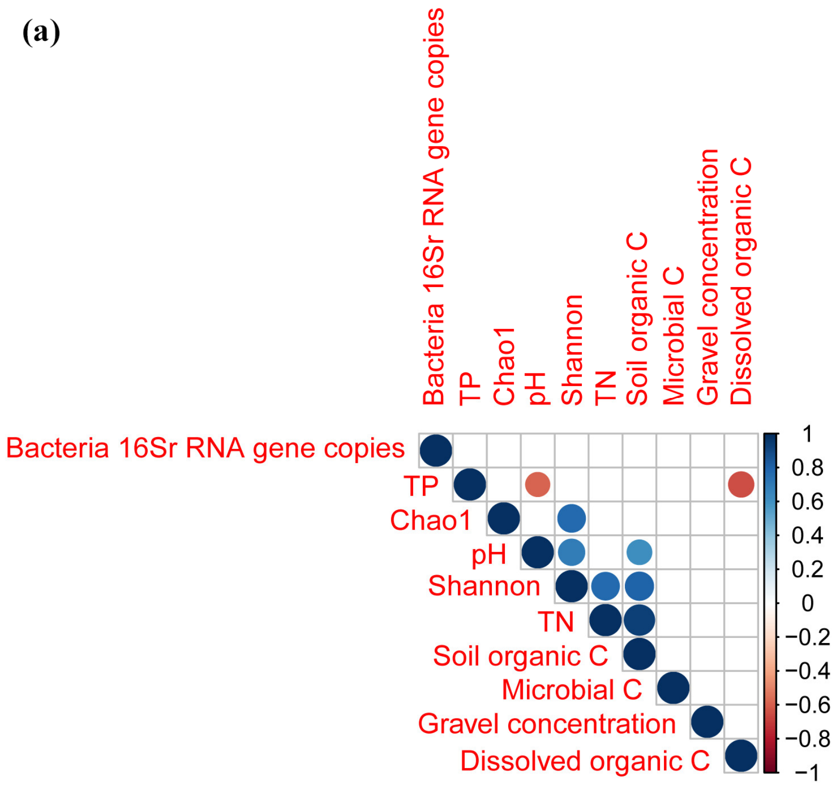

(b)

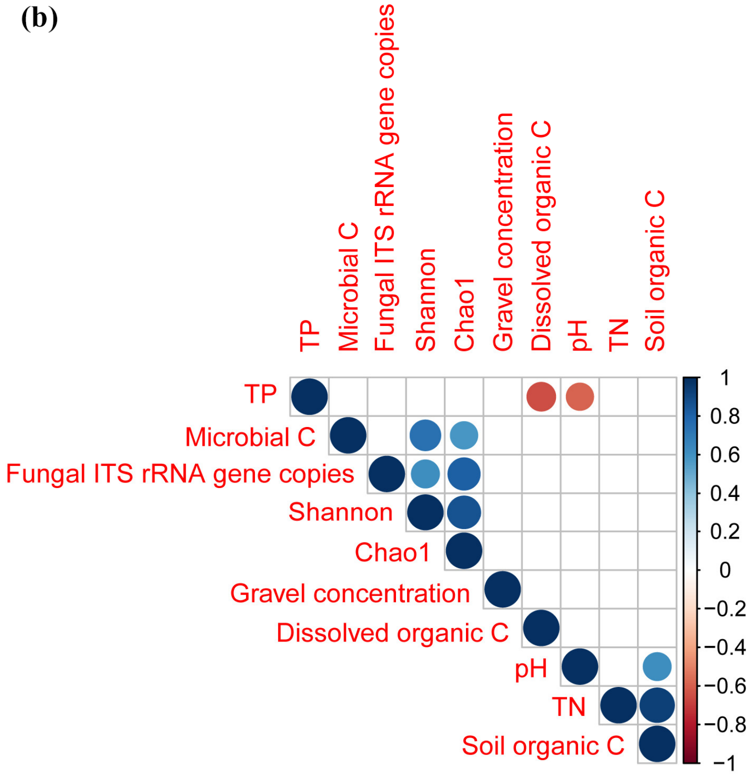

Figure S3. Pearson correlations between bacteria (a) and fungi (b) diversity index and soil physicochemical characteristics under different soil and water conservation measures.

Table S1. Normal distribution test (Shapiro–Wilkes test)

Data index	P-value
**Bacteria 16S rRNA**	**0.181**
**Fungal ITS rRNA**	**0.091**
**DOC**	**0.438**
**MBC**	**0.655**
**TP**	**0.070**
**TN**	**0.410**
**pH**	**0.080**
**SOC**	**0.327**
**GC**	**0.396**
